# Advances in the study of the effects of gut microflora on microglia in Alzheimer’s disease

**DOI:** 10.3389/fnmol.2023.1295916

**Published:** 2023-11-30

**Authors:** Jin-Jing Wu, Zhe Wei

**Affiliations:** ^1^School of Medicine, Hangzhou Normal University, Hangzhou, Zhejiang, China; ^2^School of Medicine, Lishui University, Lishui, Zhejiang, China; ^3^Institute of Breast Oncology, Lishui University Medical College, Lishui, Zhejiang, China

**Keywords:** microglia, Alzheimer’s disease, gastrointestinal microbiome, brain-gut axis, neurogenic inflammation

## Abstract

Alzheimer’s disease (AD) is a central nervous system (CNS) degenerative disorder, is caused by various factors including β-amyloid toxicity, hyperphosphorylation of tau protein, oxidative stress, and others. The dysfunction of microglia has been associated with the onset and advancement of different neurodevelopmental and neurodegenerative disorders, such as AD. The gut of mammals harbors a vast and complex population of microorganisms, commonly referred to as the microbiota. There’s a growing recognition that these gut microbes are intrinsically intertwined with mammalian physiology. Through the circulation of metabolites, they establish metabolic symbiosis, enhance immune function, and establish communication with different remote cells, including those in the brain. The gut microbiome plays a crucial part in influencing the development and performance of microglia, as indicated by recent preclinical studies. Dysbiosis of the intestinal flora leads to alterations in the microglia transcriptome that regulate the interconversion of microglia subtypes. This conversation explores recent research that clarifies how gut bacteria, their byproducts, and harmful elements affect the activation and characteristics of microglia. This understanding opens doors to innovative microbial-based therapeutic strategies for early identification and treatment goals in AD.

## 1 Introduction

Alzheimer’s disease (AD), an age-related neurodegenerative disease, is marked by a gradual onset and clinical symptoms characterized by progressive memory decline and cognitive impairment (Goedert, 2015). The distinctive pathological hallmarks of AD encompass the presence of amyloid (Aβ) deposits within brain tissue and the development of neurofibrillary tangles caused by the excessive phosphorylation of tau proteins (Ridge et al., 2013; Surguchov et al., 2023). These structural abnormalities, along with inflammatory elements, contribute to the gradual loss of neurons in susceptible regions of the brain, ultimately culminating in AD (Goedert, 2015; Goyal et al., 2021; Jucker and Walker, 2023). Furthermore, it is widely acknowledged as a chronically neuroinflammatory disorder, with inflammation playing a pivotal role in its pathogenesis (Singh, 2022).

Microglia serves as the primary immune cells within the mammalian central nervous system (CNS), with their primary function revolves around participating in the development and maintenance of CNS homeostasis (Nayak et al., 2014). Additionally, they play an active role in regulating various CNS disorders, such as neurodegenerative disease, auto-immunity, and disorders linked to nervous system development (Cook and Prinz, 2022). In the context of neurodegenerative diseases, a fundamental pathological feature is the ongoing abnormal activation of microglia, accompanied by a subsequent neuroinflammatory response. Multiple studies have underscored the association between this aberrant microglial activation and neuroinflammation, posing a significant risk in AD (Singh, 2022).

The gut fluids are the microbial communities in the intestinal tract that have a significant effect on the body’s health and illness (Sender et al., 2016). While the gastrointestinal or enteric nervous system operates autonomously from the CNS, research has uncovered a dynamic, bidirectional communication channel linking the gut flora and the brain (Cox and Weiner, 2018). This communication occurs through both direct and indirect signaling, maintaining a fluid equilibrium among the gut, CNS, and the microbial system. This intricate interplay is commonly referred to as the “gut-brain axis” (Cho et al., 2021). In the human host, these mechanisms have the potential to enhance metabolic wellbeing, but in abnormal conditions, they may pave the way for a variety of diseases (Abdel-Haq et al., 2019).

It has been recently shown that microglia are susceptible to intestinal microbial production factors (Bostick and Mazmanian, 2022). Noticeable disparities exist in the genetic makeup and physical characteristics of microglia between the pathogen-free (SPF) mice and germ-free (GF) mice (Cook and Prinz, 2022). The interplay between intestinal microbiota and microglia appears to wield influence on the development and advancement of AD, yet the exact mechanisms of these interactions remain shrouded in uncertainty. In this review, we aim to dissect the mechanisms through which gut microbes, their metabolites, and virulence factors impact microglia activation. We will delve into the intricate communication taking place between gut microbes and microglia, with the hope that these insights will prove valuable for clinical AD research.

## 2 Microglia activation and its phenotype

Microglia serve as the frontline defenders in maintaining immune responses and ensuring bodily homeostasis (Colonna and Butovsky, 2017). They play a vital role in pruning apoptotic neurons and preserving synaptic adaptability (Lukens and Eyo, 2022).

The functional states of microglia can be discerned by observing their morphological traits and the expression of various antigens. Morphologically, microglia exhibit four distinguishable phenotypes: resting, activated, amoeboid, and dystrophic. Traditionally, the branched cells were categorized as “resting,” while amoeboid and phagocytic microglia were considered “activated” (Lier et al., 2021). However, microglia can transform both morphologically and functionally in response to diverse stimuli, a phenomenon referred to as microglia activation. Activated microglia take on distinct morphologies and express specific antigens (Leng and Edison, 2021).

**FIGURE 1:**
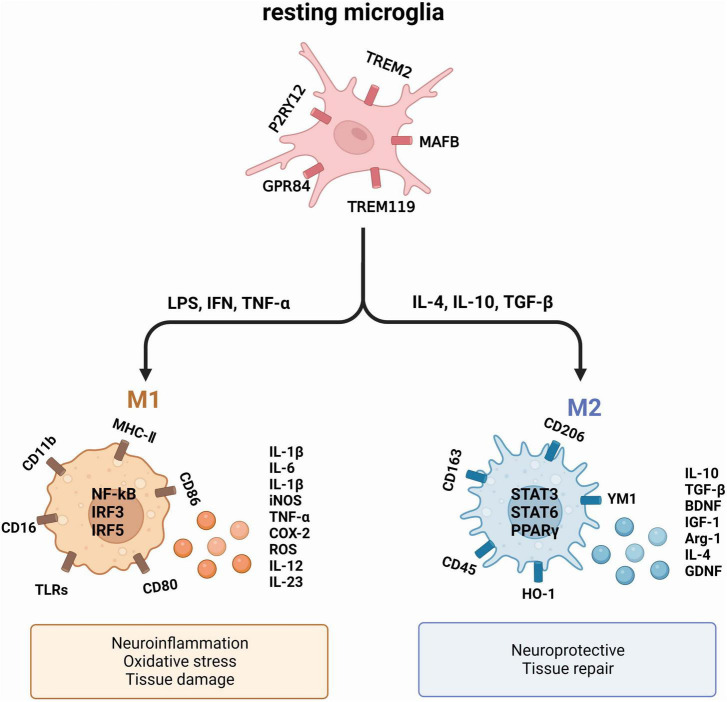
The two primary activation phenotypes of microglia. Under normal physiological conditions, microglia remain resting. However, exposure to a deleterious environment triggers the polarization of microglia into two distinct phenotypes: M1, which exhibits a pro-inflammatory nature, and M2, which showcases an anti-inflammatory nature. These phenotypes produce diverse cytokines and proteins that exert varying effects on neurons and tissues.

Numerous studies have solidly linked microglia activation to a range of brain disorders (Lull and Block, 2010; Gao et al., 2023). When microglia become activated, they churn out excessive quantities of pro-inflammatory cytokines like interleukin-1β (IL-1β) and tumor necrosis factor-α (TNF-α), which ultimately cause neurodegeneration (Al-Ghraiybah et al., 2022). Notably, in both AD patients and AD mouse models, significant spikes in the levels of pro-inflammatory cytokines such as IL-1β, interleukin-6 (IL-6), and TNF-α were observed (Reale et al., 2018; Leng and Edison, 2021). This overproduction of inflammatory cytokines further compounds the pathology of AD (Sun et al., 2020; Figure 1).

Upon activation, microglia typically undergo polarization into two distinct phenotypes: the pro-inflammatory M1 and the anti-inflammatory M2 (Sica and Mantovani, 2012; Tang and Le, 2016). M1 exert their influence on neuronal health and function by generating pro-inflammatory mediators such as TNF-α, IL-1β, IL-6, and nitric oxide synthase (iNOS), all of which contribute to neuronal harm (Song and Colonna, 2018). In comparison, M2 microglia produce substances that have a crucial function in promoting neuronal growth and safeguarding against neurological harm, which encompasses arginase-1 (Arg1) and IL-10, known for their anti-inflammatory properties (Saijo et al., 2013). The M1/M2 polarization or repolarization imbalance in microglia is mainly associated with infections, auto-immune diseases and inflammation (Wei et al., 2023).

In recent years, microglia have gained attention in AD research, particularly a microglial phenotype known as disease-associated microglia (DAM) (Paolicelli et al., 2022). Keren-Shaul et al. (2017) used advanced single-cell RNA sequencing techniques to demonstrate significant gene expression differences between DAM and steady-state microglia in rodents. The findings indicated that DAM exhibited a decrease in the expression of homeostatic genes (*p2ry12*, *trem119*, and *cx3cr1*) while showing an increase in CD74 and CD68 expression. Rangaraju et al. (2018) identified pro-inflammatory DAM isoforms (CD44 + Kv1.3 +), as well as anti-inflammatory DAM isoforms (CXCR4 + LXRα/β +) by analyzing microglia in a mouse model. In the AD model, the appearance of pro-inflammatory DAM occurred earlier than anti-inflammatory DAM, expressing pro-inflammatory genes (*tlr2*, *ptgs2*, and *il1b*), whereas anti-inflammatory DAM exhibited the expression of *apoe*, *cxcr4*, and *atf1*. Thus, microglia can interconvert between pro-inflammatory and anti-inflammatory phenotypes and, through modulation of their phenotype, the progression of AD can be delayed by inhibiting Aβ deposition, tau protein hyperphosphorylation.

## 3 Dysbiosis of the gut and Alzheimer’s disease

Dysbiosis in the gut microbiota disrupts the integrity of the intestinal mucosal barrier, elevating its permeability (Janeiro et al., 2022; Carloni and Rescigno, 2023). As a result, this causes the discharge of microbial metabolites and virulence factors into the bloodstream (Leblhuber et al., 2021). In instances where the blood-brain barrier is compromised, various pro-inflammatory cytokines circulating in the bloodstream have the potential to initiate neuroinflammation by activating microglia (Erny et al., 2015; Huang et al., 2023). This, in turn, can contribute to the development of diseases such as AD among others (Zou et al., 2022). An increasing body of recent research has highlighted a crucial connection between gut microbiota and the progression of AD. While the precise pathogenesis of AD remains elusive, there is mounting evidence suggesting that disruptions in the balance of gut bacteria could affect the creation of Aβ plaques and harm neurons through the brain-gut connection, thus playing a role in the progression of AD.

A cross-sectional study conducted in Japan discovered that *Bacteroides*’ prevalence was raised among individuals with mild cognitive impairment (MCI) without dementia. This increase in *Bacteroides* was also linked to cortical and hippocampal atrophy. Significantly, this research determined that the prevalence of *Bacteroides* was autonomously linked to these results, regardless of the existence of MCI (Saji et al., 2019). In a separate study carried out in Kazakhstan, notable variances in the makeup of gut microbiota were observed when contrasting individuals with AD to elderly individuals in good health. These variations were apparent across different taxonomic categories, encompassing phylum, class, order, and genus. Significantly, at the phylum level, there was a higher proportion of *Acidobacteriota*, *Verrucomicrobiota*, *Planctomycetota*, and *Synergistota taxa*. Within the group of bacterial genera, these differences were marked by a decrease in the proportion of various taxa such as *Bifidobacterium*, *Clostridia bacterium*, *Castellaniella*, *Roseburia*, *Tuzzerella*, *Lactobacillaceae*, and *Monoglobus* (Kaiyrlykyzy et al., 2022). All of these studies suggest that the gut microbiota is altered in AD. Therefore, studies of the gut microbiota may provide new avenues for the development of diagnostic biomarkers and therapeutic targets for AD (Table 1).

**TABLE 1:** Changes in the intestinal flora of human AD patients as compared to controls.

Bacteria altered	Commensal diversity	Sample size and location	References
At the phylum level, the AD group exhibited a decrease in *Firmicutes* and a decrease in *Actinobacteria*, and an increase in *Bacteroidetes*, compared to the controls.	AD had significantly decreased commensal diversity as indicated by reduced Shannon index	*N* = 50, 25 AD, 25 healthy controls, United States	Vogt et al., 2017
At the phylum level, the relative abundance of the *Acidobacteriota*, *Verrucomicrobiota*, *Planctomycetota*, and *Synergistota* taxa was increased in the flora of AD patients.	Examination of α-diversity through the application of Shannon, Simpson, Chao1, observed, ACE, and Fisher indices did not uncover noteworthy distinctions between individuals with AD and their healthy counterparts.	*N* = 84, 41 AD, 43 healthy controls, Kazakhstan	Kaiyrlykyzy et al., 2022
Patients with MCI had a high incidence of *Bacteroides*.	–	*N* = 82, 61 MCI, 21 healthy controls, Japan	Saji et al., 2019
In the AD group, the *Firmicutes* decreased and the *Proteobacteria* increased, with the *Clostridiaceae*, *Lachnospiraceae*, and *Ruminococcaceae* decreasing.	AD had significantly decreased commensal diversity as indicated by reduced Shannon index, and the Simpson index	*N* = 97, 33 AD, 32 aMCI, 32 healthy controls, China	Liu et al., 2019
The fecal microbial composition of AD patients was quite distinct from HC. *Bifidobacterium*, *Sphingomonas*, *Lactobacillus*, and *Blautia* were enriched, while *Odoribacter*, *Anaerobacterium*, and *Papillibacter* were reduced.	The richness of gut microbiota in the AD group was reduced	*N* = 92, 60 AD, 32 healthy controls, China	Zhou et al., 2021

In a separate animal study (Chen et al., 2020), it was observed that distinctions in microbial community composition emerged before the onset of Aβ plaque formation and neuroinflammation in the cerebral cortex of both WT mice and APP/PS1 mice. Furthermore, as these mice aged, there was a notable transition in their gut microbiota toward a profile associated with inflammation, characterized by an increase in the abundance of *Escherichia-Shigella* and *Desulfovibrio*. At the site of Aβ deposition in the brain, there was a significant build-up of microglia during this transition (Zhang et al., 2017). The results indicate that alterations in the gut microbiome may occur before the development of key pathological features of AD, ranging from the accumulation of Aβ plaques to inflammation in the hippocampus and prefrontal cortex of the brain (Goyal et al., 2021; Huang et al., 2023). Collectively, studies have underscored the connection between gut microbiota alterations and the neuroinflammatory aspects of AD pathology (Thu Thuy Nguyen and Endres, 2022). Consequently, dysbiosis of the gut flora holds promise as a significant diagnostic biomarker and a potential therapeutic target for AD.

## 4 Regulation of microglia activation and function by intestinal microbes

**FIGURE 2:**
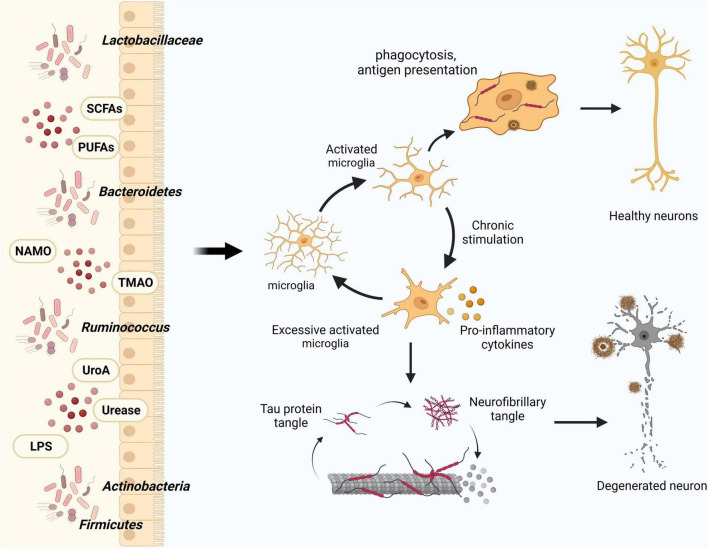
Influence of gut flora and metabolites on microglia in modulating AD pathology. Intestinal microbes and their metabolites play a crucial role in maintaining central nervous system homeostasis by modulating microglial activation, thereby influencing the clearance of Aβ and Tau proteins. In the course of AD progression, increased activation of microglia leads to the generation of pro-inflammatory factors that detrimentally affect neurons and their synaptic connections.

In the context of dysbiosis of the intestinal flora and its effect on disease progression, microglia serve as vital intermediaries. An animal experiment demonstrated that modulating the constitutive or inducible microbiota in *5* × *FAD* transgenic mice has different effects on the microglial Aβ clearance processes, ultimately mitigating neurodegeneration and cognitive impairment (Mezö et al., 2020). In their study, Huang et al. (2023) employed single-cell nucleus sequencing to unveil how intestinal microbes regulate the transformation of microglial subtypes. Furthermore, they experimentally validated that microbial colonization could rectify the majority of the altered differentially expressed genes (DEGs) in microglia, encompassing classical genes like *apoe* and *trem2*. Gut microbes can indirectly impact the formation of microglial subtypes. Therefore, investigating the precise mechanisms through which intestinal microbes exert influence on microglial activation and phenotypic modulation, via the manipulation of intestinal microbes, could provide novel perspectives on the development of AD (Figure 2).

### 4.1 Effect of intestinal microbes on microglia activation

#### 4.1.1 Bacteroides

The gut microbiome exhibits correlations with the expression of microglial markers. One study identified a connection between the abundance of *Bacteroides* in individuals with Parkinson’s disease and markers of inflammation in their blood, as well as motor impairment (Bartl et al., 2022). Another investigation demonstrated that the repetitive usage of colony-stimulating factor 1 receptor inhibitor (PLX5622) led to the elimination of microglia in adult mice, resulting in irregularities in the composition of their intestinal flora. Additionally, transcript levels of microglia markers such as *tgfb1*, *aif1*, and *csflr* in the prefrontal cortex of these mice appeared to be positively associated with the relative abundance of *Bacteroides caecimuris* (Yang et al., 2022). Furthermore, the *Enterobacteriaceae* genus exhibited an association with a high-fat diet (HFD). Metagenomic analysis revealed an elevated presence of *Bacteroides* and a reduced presence of *S24-7* in the gut of the HFD group. Simultaneously, there was a decline in the claudin-5 protein found in the intestinal tight junctions, while the area occupied by microglia increased. The research revealed that *Bacteroides* had the ability to generate a fragilysin toxin which disrupted the parietal epithelial barrier by degrading the extracellular structural region of E-cadherin on intestinal cells using proteases, leading to the disassembly of the junction (Chompre et al., 2022). Collectively, studies indicate that *Bacteroides* may activate microglia through the C/EBPβ/AEP pathway and release pro-inflammatory cytokines including IL-6 and IL-1β (Xia et al., 2023), although the exact mechanism behind this effect is still unknown.

#### 4.1.2 Ruminococcus

Jang et al. (2018) found that *Ruminococcus* spp. abundance was significantly elevated in the intestinal flora of immune-tolerant mice, expression of the tight junction protein claudin-5 was significantly elevated, showed lower concentration of IL-1β, and microglia were M2-polarized through a study of an epileptic mouse model. It was also hypothesized that *Ruminococcus* spp. could reduce intestinal mucosal permeability and promote microglia M2 polarization. However, in another study (Teng et al., 2022), the *Ruminococcaceae* metabolite isoamylamine (IAA) was found to be enriched in aged mice and the elderly, and young mice given IAA orally exhibited age-related cognitive dysfunction. Further studies revealed that the gastric *Ruminococcus* metabolite IAA induces microglia apoptosis by recruiting the transcriptional regulator p53 to the S100A8 promoter region and that overexpression of S100A8 induces division of caspase-3, a marker of apoptotic cells. Gut microbes have the potential to impact microglia activation through the generation of diverse metabolites.

#### 4.1.3 Lactobacillus

Numerous studies have demonstrated that *Lactobacillus* can stimulate cytokine production in microglia/macrophages and induce the polarization of microglia/macrophages into various phenotypes through multiple pathways (Christensen et al., 2002; Lebovitz et al., 2018). This in turn diminishes the inflammatory response and promotes the generation of anti-inflammatory microglia. Lebovitz et al. (2019) reported that maternal microbiota dysbiosis (MMD) caused microglia activation and the upregulation of *cx3cr1*. This upregulation was associated with enhanced expression of the senescence-related genes *il1*β and *trp53*. Subsequently, they demonstrated that the existence of a new intestinal commensal variant, *Lactobacillus murinus* (HU-1), reverses microglial activation. In an animal study, it was observed that *Lactobacillus rhamnosus* (LGG-CM) inhibited the NF-κB pathway by reducing IκBα phosphorylation. This facilitated the polarization of microglia/macrophages toward M2 and suppressed polarization toward M1 (Lin et al., 2021). Another study found that the lifespan of the model organism *C. elegans* could be regulated by administering Probio-M9, a *Lactobacillus rhamnosus* bacterium isolated from healthy human breast milk. This regulation was linked to the p38 cascade and the daf-2 signaling pathway (Zhang et al., 2022). Likewise, Duan et al. (2021) discovered that supplementation with *Lactobacillus reuteri (L. reuteri)* and butyrate reversed the activation of microglia and dendritic spine loss in mice subjected to an HFD. They postulated that *Lactobacillus royal* might either secrete butyrate or modulate the intestinal flora to enhance butyrate production, thereby influencing microglia activation.

### 4.2 Effect of gut microbial-derived metabolites on microglia activation

**FIGURE 3:**
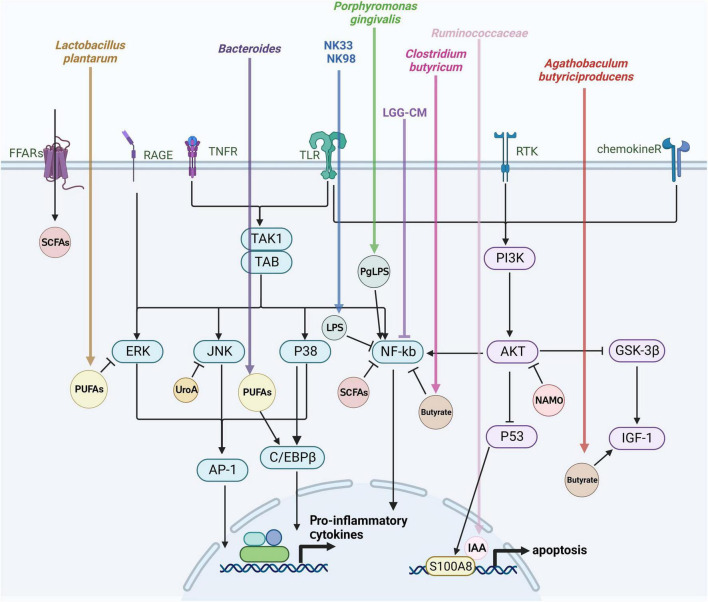
Mechanisms of gut flora-derived metabolite regulation of microglia activation. Microglia activation is closely tied to various signaling pathways, including extracellular signal-regulated protein kinases (ERK), c-Jun N-terminal kinase (JNK), P38 mitogen-activated protein kinase (P38 MAPK), protein kinase B (AKT), and the nuclear factor-κB (NF-κB) pathway. This figure outlines the effects of gut microbes and their metabolites on microglia through the aforementioned signaling pathways discussed in this review.

The gut microbiota plays an essential regulatory role in the brain-gut axis, with this regulation hinging on microbiota-produced metabolites and how they interact with the cells of the host. The interactions have the ability to either trigger or block signaling pathways, ultimately influencing the host’s health in either a positive or negative manner. Bacterial metabolites that are engaged in these relationships include by-products of bacterial metabolism, such as short-chain fatty acids (SCFAs). The composition of microorganisms determines the abundance and availability of metabolites, which are regulated by dietary and environmental factors (De Vos et al., 2022). Investigating the mechanisms through which gut microbial metabolites regulate microglia activation can enhance our comprehension of the disease progression in AD. The subsequent discussion centers on the effects of gut flora metabolites on microglia activation (Figure 3).

#### 4.2.1 SCFAs

Wang et al. (2021) utilized existing databases to analyze Microbial Metabolite-Gene-Pathway-Phenotype (MGPPN) data, leading to the conclusion that there is a significant correlation between microglial functional and physiological dysfunction and AD. This suggests a strong association between AD, microglia, and gut microbes, particularly emphasizing the significant correlation with SCFAs like acetic acid, propionic acid, and butyric acid. These SCFAs are metabolites produced from the fermentation of dietary fiber by bacteria. *In vitro* studies have revealed that SCFAs directly impact microglia through a diffusion mechanism, leading to the inhibition of histone deacetylase activities, NF-κB activities, and inflammation induced by lipopolysaccharides (LPS). This impact is independent of the known SCFA receptor and monocarboxylate transporter, Mct1 (Caetano-Silva et al., 2023). However, Colombo et al. (2021) discovered that SCFAs may actually enhance Aβ deposition by activating microglia through the upregulation of *apoe* gene expression. It seems that the biological effects of SCFAs on microglia are heavily contingent on specific disease conditions. Consequently, the precise mechanisms through which SCFAs regulate microglia remain to be explored, particularly in relation to distinct mouse models of AD or in individuals.

Butyrate, a type of SCFA, is primarily produced through bacterial fermentation of colonic fiber and has been extensively examined as a histone deacetylase inhibitor in pharmacological research. Research has shown that butyrate mitigates the increase in pro-inflammatory cytokine production in microglial cells drived from elderly mice (Matt et al., 2018). Furthermore, administering butyric acid-producing *Clostridium butyricum* (CB) to APP/PS1 mice prevented microglia activation, cognitive impairments, Aβ deposition, and the release of TNF-α and IL-1β in the cerebrum, while concurrently rectifying dysbiosis in the intestinal flora. Additional research uncovered that butyrate treatment inhibited the activation of microglia by suppressing NF-κB p65 phosphorylation in Aβ-induced BV2 microglia and reducing CD11b and COX-2 levels in cells (Sun et al., 2020). In a study involving mice with chronic alcohol-induced impairment, Wei et al. (2023) discovered that administration of sodium butyrate (NaB) modified the microglial polarity imbalance (M1/M2) by interacting with GPR109A, leading to upregulated expression of PPAR-γ and downregulated activation of TLR4/NF-κB. Furthermore, they conducted sequencing and analysis of mouse gut bacteria, revealing that following NaB administration, there was a reduction in the *Bifidobacterium*, *Bacteroides*, and *Lactobacillus* genera, while the *Parvibacter*, *Faecalibaculum*, and *Alloprevotella* genus experienced an increase at the genus level. In a separate animal experiment, *Agathobaculum butyriciproducens* (SR79), a severely anaerobic bacterium producing butyric acid, was utilized. SR79 enhanced cognitive function and ameliorated AD pathology in animal models by modulating neuroinflammation and IGF-1 signaling (Go et al., 2021).

Acetic acid, a SCFAs with the capability to cross the blood-brain barrier, influences microglial function within the brain. Acetate regulates microglia’s maturation and metabolic state, thereby impacting their responses to inflammation (Frost et al., 2014). Research has revealed that acetic acid inhibited nitric oxide (NO) generation in the primary rat microglial cells, thus exerting a neuroprotective effect. However, this effect is not observed in BV-2 microglial cells, suggesting differential responses to acetic acid among microglia of varying origins. The authors propose that acetate’s mechanism of action on microglia likely involves the suppression of the mitochondrial respiratory chain complex, which plays a role in regulating iNOS activity (Moriyama et al., 2021). Additionally, in another study, the authors found that acetate exhibited a dual role in microglial TNF-α generation. It could both promote and suppress TNF-α secretion, depending on the acetate concentration and the activation state of the microglial cells. The authors concluded that acetate facilitated access to chromatin, histone acetylation, and TNF-α production in LPS-stimulated microglial cells (Fragas et al., 2022).

#### 4.2.2 Polyunsaturated fatty acids (PUFAs)

During a clinical trial, daily high-dose supplementation of mixed omega-3 polyunsaturated fatty acids (PUFAs) for 8 weeks significantly increased the abundance of *Bifidobacterium*, *Roseburia*, and *Lachnospira* species, while decreasing the presence of Coprococcus and Faecalibacterium (Watson et al., 2018). Another study, focusing on microglial size, revealed that PUFAs reduced the baseline activation state of microglia. This was evidenced by larger cell sizes, reduced cell numbers, and lower labeling intensity. Additionally, the PUFAs diet positively influenced the composition of the intestinal bacterial flora (Hakimian et al., 2019). Moreover, Chen et al. (2022) observed that in *3* × *Tg* AD transgenic mice, microglia activation and inflammation levels were notably lower than in germ-positive mice. In contrast, transplanting gut microbiota from AD patients exacerbated microglial activation and inflammatory responses in 3 × Tg mice compared to microbiota transplants from healthy donors. The authors further found that the gut flora could influence the levels of PUFAs metabolites and their oxidative enzymes, subsequently modulating the C/EBPβ/aspartate endopeptidase (AEP) signaling pathway within microglia. This modulation led to the promotion of neuronal degenerative changes and cognitive dysfunction through the cleavage of Tau proteins. They also discovered an increase in *Bacteroides*, which mediate the metabolism of proinflammatory PUFAs, in the gut microbiomes of AD patients. This increase plays a role in regulating the activation of microglia in the brain. The administration of PGE2-G, a metabolite produced from AA by the bacterial strain *Bacteroides*, has been found to restore AD pathologies and microglial activation in GF mice, especially in the presence of SCFA. Additionally, the gut lactic acid bacterium, *Lactobacillus plantarum*, produces two long-chain fatty acids: 10-oxo-*trans-*11-octadecenoic acid (KetoC) and 10-hydroxy-*cis-*12-octadecenoic acid (HYA), derived from linoleic acid. These compounds possess anti-inflammatory properties by inhibiting the production of nitric oxide (NO) and the expression of iNOS in BV2 microglia when stimulated by LPS. This anti-inflammatory mechanism potentially functions by inhibiting ERK phosphorylation, with a hypothesis that this inhibition might be associated with a decrease in iNOS transcription, which is facilitated by transcription factors like AP-1 (Ikeguchi et al., 2018).

#### 4.2.3 Trimethylamine N-oxide

Trimethylamine N-oxide (TMAO) is a metabolite produced by the gut microbiota from specific dietary nutrients and is linked to cardiovascular and neurological disorders. Imbalances in the gut microbiota can result in elevated levels of circulating TMAO (Meng et al., 2019).

According to clinical research, individuals with MCI and AD exhibit elevated levels of TMAO in their cerebrospinal fluid (CSF) compared to those without cognitive impairment. Elevated CSF TMAO has also been linked to Aβ42, a biomarker of AD pathology, and neurofilament light chain protein, which indicates neuronal degeneration (Vogt et al., 2018). Another study conducted on mid-aged male *C57BL/6* mice, 24 and 72 h after a sham operation, observed that a choline-enriched diet significantly elevated serum TMAO levels. This diet also increased the activation of microglia and astrocytes surrounding the hematoma compared to a regular diet. However, TMAO did not impact the expression of *p38 mapk*, *myd88*, *hmgb1*, or *il-1*β, and the precise mechanism of its activation remains unknown (Li et al., 2022). In animal experiments, conducted by Meng et al. (2019), elevated circulating TMAO levels were found to contribute to cognitive decline. This was associated with increased microglia activity, neural inflammation, and the generation of reactive oxygen species (ROS) in the hippocampus of surgically treated rats. Furthermore, elevated TMAO levels were shown to down-regulate the hippocampal antioxidant enzyme, methionine sulfoxide reductase (MsrA), potentially increasing vulnerability to surgically induced oxidative stress, ultimately leading to heightened neural inflammation and post-operative cognitive decline in aged rats.

#### 4.2.4 Nicotinamide n-oxide (NAMO)

Nicotinamide nitrogen oxide (NAMO), an oxidized derivative of nicotinamide, is primarily synthesized by certain intestinal bacteria, including *Lactobacillus gasseri* and *Lactobacillus reuteri.* NAMO plays a crucial role in regulating microglia activation. Research by Li et al. (2023) has shown that NAMO, as an intestinal metabolite, promotes the production of nicotinamide adenine dinucleotide (NAD +) and facilitates mitophagy, a process that clears damaged mitochondria. This in turn inhibits microglia activation and the subsequent inflammatory responses. Nicotinamide riboside (NR), a precursor to nicotinamide adenine dinucleotide (NAD +), a form of vitamin B3, has demonstrated promising effects. In a separate investigation, NR was discovered to decrease the activation of microglia in mice exhibiting depression-like symptoms induced by alcohol consumption. Furthermore, NR lowered the levels of both pro-inflammatory (IL-1β, IL-6, and TNF-α) and anti-inflammatory (IL-10 and TGF-β) cytokines in the brains of these mice. NR also notably up-regulated the expression of brain-derived neurotrophic factor (BDNF) and alleviated the inhibition of the AKT/GSK3/β-catenin signaling pathway in the hippocampus. These findings demonstrate the efficacy of NR treatment in preventing excessive microglia activation (Jiang et al., 2020).

#### 4.2.5 Urolithin

Extensive studies have been carried out on urolithin, the gut metabolite of red raspberry polyphenols. Numerous studies have consistently highlighted its potential to reduce both inflammation and oxidative stress (Espín et al., 2013; Kujawska et al., 2019). Nevertheless, the exact mechanism behind these effects is yet to be completely understood. In a study by Toney et al. (2020), urolithin A (UroA), derived from the gut metabolism of red raspberry polyphenols (RRW), was found to not only decrease the induction of iNOS gene expression but also promote the polarization of M2 microglia. This dual action is achieved by suppressing exposure to pro-inflammatory cytokines and modulating the JNK/c-Jun signaling pathway. Another *in vitro* study revealed that Urolithins effectively reduced the levels of NO, IL-6, prostaglandin E2, and TNF-α in the media of LPS-stimulated BV-2 microglial cells. Moreover, Urolithins demonstrated a capacity to mitigate apoptosis and the release of caspases 3/7 and caspases 9 in H_2_O_2_-induced oxidative stress of BV-2 microglial and SH-SY5Y neuron cells. In summary, these discoveries propose that urolithin could potentially provide protection to the nervous system by blocking caspase activation when cells undergo apoptosis due to oxidative stress (DaSilva et al., 2019).

#### 4.2.6 Ureases

*Proteus mirabilis*, a gram-negative rod-shaped bacterium commonly found in the intestinal microbiota, synthesizes a urea-inducible urease (PMU), known as a factor contributing to its virulence (Armbruster et al., 2018). Investigation of PMU’s non-enzymatic activities in human and mouse cell models revealed its potential to induce platelet aggregation, elevate intracellular calcium ions, and generate ROS. *In vitro* experiments with microglia BV-2 cells indicated that PMU could stimulate the release of pro-inflammatory cytokines IL-1β and TNF-α, suggesting its neurotoxic and neuroinflammatory effects on microglia. These results indicated that PMU, in addition to its primary enzymatic function of catalyzing urea breakdown to produce ammonia, possesses non-enzymatic activities associated with pathogenicity, including pro-inflammatory and neurotoxic effects (Grahl et al., 2021).

*Helicobacter pylori’s urease* (HPU), a cellular protein, was studied *in vitro* by Uberti et al. (2022). Their research showed that HPU reduced the viability of SH-SY5Y neuroblastoma cells and BV-2 microglia, triggered the generation of ROS, elevated intracellular calcium ion levels, and induced the release of the inflammatory factors IL-1β and TNF-α. In animal experiments, rats subjected to daily intraperitoneal injection of HPU (5 micrograms) for 7 days displayed hyperphosphorylated tau protein in hippocampal tissue and an overexpression of Iba1, a marker of microglial activation. Behavioral assessments demonstrated cognitive impairments in object recognition and elevated cross-maze tests among HPU-treated rats. These findings suggest the possibility that *Helicobacter pylori* infection could exacerbate AD pathology through its urease-mediated tau protein pathology (Hanger et al., 2009).

#### 4.2.7 Lipopolysaccharide (LPS)

Numerous studies consistently demonstrate that bacterial-derived LPS activates microglia, prompting the production of inflammatory mediators and cytokines, including NO, IL-6, TNF-α, and PGE2 (Z. Chen et al., 2012; Zhao et al., 2022). These inflammatory biomarkers is crucial in the development of AD by instigating the production of aberrant proteins, including tau protein, Aβ, and α-synuclein (Ju Hwang et al., 2019; Kim et al., 2021). This cascade ultimately activates the apoptotic pathway (caspase activation), leading to a proinflammatory environment and microglial proliferation (DaSilva et al., 2019). Clinical investigations have revealed the presence of Gram-negative bacterial LPS, *E. coli K99* hair protein, and DNA in the brains of both control and AD groups. However, the AD group exhibited elevated levels of *K99* and LPS compared to the control group. Furthermore, it was observed that LPS co-localized with Aβ1-40/42 within Aβ plaques and the perivascular region of the AD brain. These findings imply the relevance of Gram-negative bacteria to the pathology of AD (Zhan et al., 2016). In another investigation, it was discovered that long-term systemic exposure to *Porphyromonas gingivalis* (PgLPS) led to cognitive deterioration in mice of middle age. This effect was attributed to the actions of IL-6 and IL-17, which are common risk factors. Additionally, PgLPS was found to be involved in the accumulation of Aβ in neurons via NF-κB translocation. Moreover, there was an increased expression of IL-17 in microglial cells (Gu et al., 2020). Furthermore, *Lactobacillus reuteri* NK33 and *Bifidobacterium adolescentis* NK98 were found to inhibit microglia activation by modulating the entry of LPS into the brain and suppressing the activation of NF-κB and the expression of IL-6 in LPS-treated BV-2 cells (Jang et al., 2019).

## 5 Strategies for the treatment of AD based on gut microbes

### 5.1 Fecal microbiota transplantation (FMT)

The research unveiled that Fecal Microbiota Transplantation (FMT) caused a shift in the gut microbial makeup of septic patients (Khoruts and Weingarden, 2014). In septic mice, it enhanced spatial learning and memory, curbing microglia activation in the cortex and attenuated levels of IL-1β, IL-6, TNF-α. Intriguingly, severing the vagus nerve reversed the altered gut microbial composition (Li et al., 2018). In another study, FMT reduced microglia and astrocyte activation in a mouse model of Parkinson’s disease, corrected gut microbial imbalance, and was neuroprotective by reducing the TLR4/TBK1/NF-κB/TNF-α signaling pathway in both the gut and brain (Sun et al., 2018).

### 5.2 Dietary treatment

A diet rich in fermentable fiber tends to shifts the production of SCFAs in favor of butyric acid (Yang et al., 2020). When examining the distal colon, older animals consuming a low-fiber diet showed increased inflammatory infiltration, while those on a HFD exhibited significantly reduced inflammatory infiltration. Moreover, adult mice on an HFD showed elevated DNA methylation (Chompre et al., 2022). Interestingly, a negative correlation emerged between the expression of pro-inflammatory factors in microglia and the concentration of butyric acid, acetic acid, and total SCFAs in the cecum (Matt et al., 2018). Additionally, the inclusion of soluble fiber in the diet led to higher SCFAs levels in the cecum and hepatic portal vein, ultimately dampening the inflammatory response in isolated primary microglia (Caetano-Silva et al., 2023).

### 5.3 Probiotics

Cui et al. (2021) discovered that probiotic treatment effectively restrained the activation of hippocampal microglia triggered by fear conditioning. Notably, this treatment altered the gut microbiome by reducing the proportion of *Ruminiclostridium* and *Allobaculum* while increasing the prevalence of immobile bacilli, thereby hastening the disappearance of fear. In the case of rats fed a HFD, the intake of prebiotics, probiotics, or a synbiotic regimen all demonstrated significant reductions in hippocampal oxidative stress and apoptosis, which also reduced microglia activation and led to recovery of cognitive function (Yu et al., 2015). Furthermore, it is suggested that these neuroprotective effects could potentially be mediated through various pathways, including the mitigation of inflammation, decreased hippocampal oxidative stress, reduced hippocampal apoptosis, mitigation of mitochondrial dysfunction, and correction of microglial cell dysfunction (Chunchai et al., 2016). However, it’s important to note that the positive impacts of probiotics, prebiotics, or symbiotic bacteria are strain-specific (D’Argenio and Sarnataro, 2021). Additional research is necessary to gain a deeper comprehension of the mechanisms that govern the function of microglia in cognitive processes and the communication pathways between glial cells (Chunchai et al., 2018).

## 6 Conclusion

So far, research on how microbiota can affect microglia has revealed a number of phenomena that could be relevant to neurological disorders. Yet, it remains uncertain whether the changes observed in microbial communities associated with diseases are the cause or result of altered brain function. It is also uncertain if interventions targeting the microbiome can improve microglial function and produce positive outcomes in neurodegenerative disorders. The gut microbiota can send signals to the brain that can either improve or worsen the progression of diseases. These changes may take place through gut-mediated alterations in microglial cell behavior (Abdel-Haq et al., 2019). This paper provides a summary of the mechanisms through which intestinal microbes and their byproducts, as well as virulence factors, influence microglia and impact the onset and progression of AD. This comprehension has the potential to facilitate early detection and the creation of novel treatment objectives and medications for addressing AD.

## Author contributions

J-JW: Writing – original draft. ZW: Writing – review and editing.
